# Electronic Properties of DNA Origami Nanostructures Revealed by *In Silico* Calculations

**DOI:** 10.1021/acs.jpcb.4c00445

**Published:** 2024-05-07

**Authors:** Busra Demir, Caglanaz Akin Gultakti, Zeynep Koker, M. P. Anantram, Ersin Emre Oren

**Affiliations:** † Department of Materials Science & Nanotechnology Engineering, TOBB University of Economics and Technology, Ankara 06560, Türkiye; ‡ Bionanodesign Laboratory, Department of Biomedical Engineering, TOBB University of Economics and Technology, Ankara 06560, Türkiye; § Department of Electrical and Computer Engineering, University of Washington, Seattle, Washington 98115, United States

## Abstract

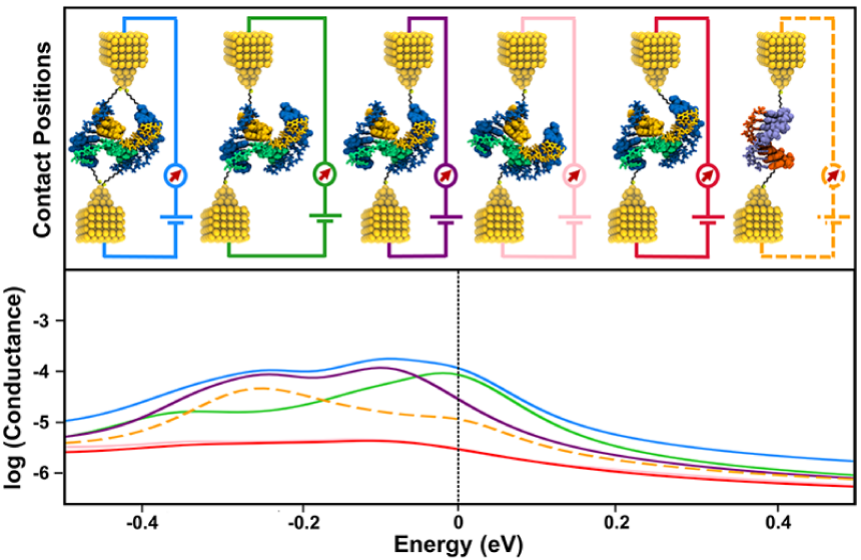

DNA origami is a pioneering approach for producing complex 2- or 3-D shapes for use in molecular electronics due to its inherent self-assembly and programmability properties. The electronic properties of DNA origami structures are not yet fully understood, limiting the potential applications. Here, we conduct a theoretical study with a combination of molecular dynamics, first-principles, and charge transmission calculations. We use four separate single strand DNAs, each having 8 bases (4 × G_4_C_4_ and 4 × A_4_T_4_), to form two different DNA nanostructures, each having two helices bundled together with one crossover. We also generated double-stranded DNAs to compare electronic properties to decipher the effects of crossovers and bundle formations. We demonstrate that density of states and band gap of DNA origami depend on its sequence and structure. The crossover regions could reduce the conductance due to a lack of available states near the HOMO level. Furthermore, we reveal that, despite having the same sequence, the two helices in the DNA origami structure could exhibit different electronic properties, and electrode position can affect the resulting conductance values. Our study provides better understanding of the electronic properties of DNA origamis and enables us to tune these properties for electronic applications such as nanowires, switches, and logic gates.

## Introduction

The field of molecular electronics uses molecules as electronic components^1^ and has intriguing applications in a variety of fields, including energy storage devices,^2^ logic circuits,^3^ optoelectronics,^4^ and sensors.^5^ Molecular electronics offer various fundamental advantages.^6^ First, the size of molecules used is in the realm of nanometers, and thus the device packing densities can be increased with lower cost, high efficiency, and power dissipation benefits. Second, one can use specific intermolecular interactions to form desired geometries via self-assembly in a bottom-up fashion. Therefore, various organic and inorganic molecules have been the subject of extensive research to engineer novel electronic components.^7^

For more than two decades, DNA has been at the forefront of molecular electronics research. Besides its unique properties such as self-assembly, programmability, and biocompatibility, DNA consists of four nucleobases: adenine (A), guanine (G), cytosine (C), and thymine (T), which play a large role in determining its stability, flexibility, and polymorphic structure.^8^ These four nucleobases each have distinct energy levels, and when combined in a sequence, their electronic properties can be engineered to have a variety of interesting behaviors, such as double barrier resonant tunneling,^9^ miniband formation,^10^ and large bandgap semiconductor.^11^

Electron transport through a single DNA molecule has been broadly studied both from experimental and theoretical perspectives. These studies showed that charge can be transmitted along the DNA, having tens of nanometer length, via overlapping π–π orbitals of the stacked bases.^12^ Modeling studies of charge transport based on both the decoherent transmission^13−15^ and and hopping^16−18^ models can be found. Furthermore, DNA strands with guanine bases are known to have a higher conductivity – most likely because guanine’s highest occupied molecular orbital (HOMO) level is the closest to the electrodes’ Fermi level among the four DNA bases. Studies with single-molecule conductance measurements with break junction-based methods have demonstrated that DNA’s electrical conductance is highly sensitive to chemical modifications,^19^ conformational changes,^20,21^ single base mismatches,^22^ and the intercalation of small molecules.^15^

DNA nanotechnology studies, aiming to use DNA as molecular building blocks, began in the 1980s,^23^ and after Rothemund’s work in 2006,^24^ the creation of large DNA origami tiles on a surface has become a common practice.^25−28^ For instance, Maune et al.^29^ developed an approach for arranging two-dimensional single-walled carbon nanotubes on a SiO_2_ substrate using DNA origami templates. Liu et al.^30^ used DNA origami templates to deposit metal particles with an average height as small as 32 nm for the realization of a prototype DNA-based nanoelectronic circuit. Recently, Aryal et al.^31^ fabricated the electrically linked gold–tellurium metal–semiconductor junctions on DNA origami by using the location-specific binding of gold and tellurium nanorods. Although scientists have a reasonable understanding of a single DNA’s electronic properties, DNA origami’s electronic properties remain mostly unexplored.^32^ The following observations have to be taken into account to utilize DNA origami in molecular electronics: DNA origami structures have crossover regions (also known as Holiday Junctions), where two DNA helices bind together (Figure S1). While the crossovers provide structural stability for the entire origami geometry, they are also responsible for local distortions in the base pair stacking and thus may influence the electrical conductance. These distortions are reported to be limited to four base pairs forming the crossover region.^33^ DNA origami structure consists of several double strands connected to one another, which can potentially create different charge transport pathways.

**Figure 1:**
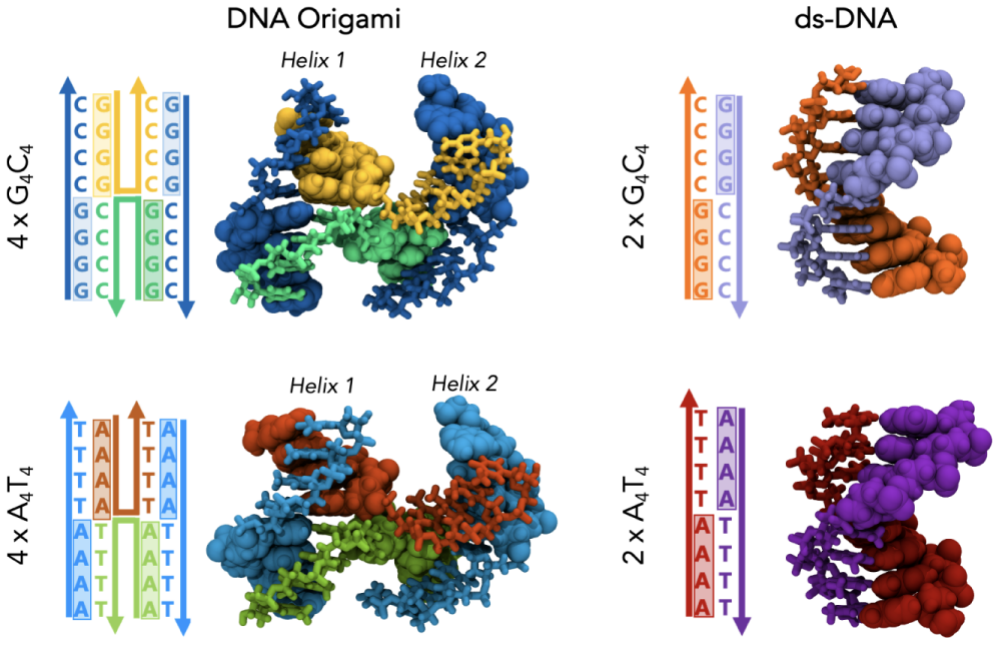
3D geometry of the representative structures, which are used for QM calculations and their sequences.

In this paper, we address the electronic properties of DNA origami structures with a combination of molecular dynamics (MD) simulations, density functional theory (DFT), and Green’s function-based charge transport (CT) calculations. We focus on the comparison of an eight-base pair long dsDNA and an eight-base pair long two helix DNA origami molecule (Figure 1). The role of these short regions is essential to understand before embarking on a study involving larger origami structures. We investigate the impact of the crossover region on the electronic properties including the density of states and charge transmission pathways. We show that compared to dsDNA with the same sequence, DNA origami structures exhibit a higher density of states and a smaller energy difference between the HOMO orbitals close to the band gap in the 4 × G_4_C_4_ sequence. We find that the crossover regions contain a high density of states primarily at lower energy levels rather than near the HOMO level, which can be attributed to the unnatural looping of the backbone atoms from one helix to another. Next, we demonstrated that the sequence affects the conductance in both dsDNA and DNA origami cases. We also reveal that the transmission also depends on the electrode contact points. In the experiments carried out via single molecule break junction (SMBJ),^20,22^ the DNA molecules are modified with thiol linkers to contact the gold electrodes. Thus, one can inject the charge locally into these atoms covalently bonded to thiol linkers. If the contacts are on two different helices, the transmission is lowered due to the increased path crossing through crossover regions. Finally, we demonstrate that new charge transport pathways can also emerge as the relative positions of the two helices of DNA origami changes.

## Theoretical Methods

### Molecular Dynamics

Eight base pair long DNA origami structures with 4 × G_4_C_4_ and 4 × A_4_T_4_ sequences are designed via caDNAno^34^ software and converted into an atomistic model using the web-based caDNAno to PDB converter tool.^35^ 8 bp dsDNA with the same sequence for each case is generated using AMBER Nucleic Acid Builder. The length of the structures is chosen because of the DFT limitations. All structures are neutralized with Na^+^ counterions and added into an octahedral water box, which had a 15 Å cutoff from the DNA molecules.

First, water molecules and counterions were subjected to 500 steps of energy minimization, while DNA molecules were restrained with 50 kcal/mol force. Then, 5000 steps of energy minimization were applied to the whole system without any restraint on any molecule. Then, the system is heated to 300 K in NVT ensemble within 100 ps, while a 50 kcal/mol restraint force was applied to the DNA molecules. Next, the system equilibrated for 100 ps, while a 0.5 kcal/mol restraint force was applied only to the DNA molecules. Finally, the entire system was simulated in an NPT ensemble for 100 ns without any restraining force applied via the AMBER 20^36^ pmemd CUDA module. For all simulations, bsc1^37^ and TIP3P^38^ force fields were used to describe DNA and water molecules as well as counterions, respectively. The particle Mesh Ewald^39^ algorithm was used for long-range electrostatic interactions, and a cutoff value of 10 Å was used for the van der Waals interactions. The simulations were performed every 2 fs and recorded every 2 ps, resulting in 50,000 conformations in each trajectory. All bonds with the hydrogen atoms were constrained using the SHAKE algorithm.^40^ MD trajectory analysis was performed using VMD’s built-in analysis tools and Python’s Pytraj library.

### Representative Structure Selection

To analyze the electronic properties of each structure, we use RMSD-based clustering algorithm within VMD software.^41^ This method categorizes the conformations of the DNA observed throughout the simulation. A cutoff value of 2.5 and 1.75 Å RMSD was chosen to cluster the DNA origami and dsDNA structures, respectively. We select the centroid structures from the most populated cluster (top cluster), which have minimum RMSD difference from the rest of the conformations within the top cluster. We then perform energy minimization to the selected structures before QM calculations to relax the residual strains of the conformations due to thermal fluctuations during MD simulations. Figure 1 shows the sequence and initial structures for all cases.

### Density Functional Theory and Charge Transport Calculations

We perform DFT calculations after removing the explicit water molecules and counterions from each structure for DFT convergence. Since we remove the counterions that neutralize the negative backbone of the DNA, we set the total charge of the DNA origami structures to −28, and dsDNAs to −14, which is equal to the number of phosphate groups of the corresponding cases (the terminal bases do not include their phosphate groups). Then, we carry out the calculations using the Gaussian 16^42^ software package with the B3LYP exchange-correlation function and 6-31G(d,p) basis set. We also use the polarizable continuum model (PCM)^43^ to model the water solvent effect in the system, implicitly. We use the Avogadro^44^ software program to plot the molecular orbitals (MOs).

Next, we carry out Green’s function-based charge transport calculations by following the methods used in our previous studies.^15,45^ From the DFT calculations, we obtain the Fock, *H*_0_, and overlap matrices, *S*_0_. Then, we use the Löwdin transformation to convert *H*_0_ into a Hamiltonian, *H*_orthogonal_, where the diagonal elements represent the energy levels at each atomic orbital, and the off-diagonal elements correspond to the coupling between the different atomic orbitals:^13^
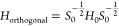
1

Since *H*_orthogonal_ has elements of each contributing basis function to define an atomic orbital, next, we partition the *H*_orthogonal_ into individual atoms and diagonalize the *H*_orthogonal_ matrix using the following transformation.
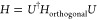
2

Here, the eigenvalues of each atom are represented by the diagonal elements of *H*, while the off-diagonal blocks indicate the hopping parameters between equivalent atoms’ molecular orbitals.

Next, we calculate the transmission and density of states (DOS) along the molecules using the Green’s function.^13^ We solve the following equation to find the retarded Green’s function (*G*^r^):
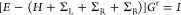
3

In eq 3, *E* is the energy, and *H* is the Hamiltonian defined in eq 2. Σ_L_ and Σ_R_ describe the left and right contact self-energies, respectively, which represent the coupling strength of the DNA molecules to the contacts through which charge is introduced and extracted from the DNA molecules. Here, both Σ_L_ and Σ_R_ are matrices of dimension *N* × *N*, where *N* is the total number of orbitals on the DNA. The only nonzero elements of these matrices correspond to atoms that make contact to the left (right) contact. All orbitals of these atoms contact the left (right) contacts. We choose , for all orbitals on atom that make contact to the left (right) contact, with off diagonal elements of Σ_L(R)_ being zero, and Γ_L(R)_ represent the energy independent coupling parameter. Similarly, we use the self-energy of the decoherence probe defined with Σ_B_, which characterizes the coupling strength between the DNA atoms and the decoherence probes. This is also defined with an energy independent parameter: at all orbitals of the DNA except those making contact to the left (right) contacts. Note that all off diagonal elements of Σ_B_ are zero. Here, Γ_B_ represents the energy independent coupling parameter for Büttiker probes. We set the left (right) Γ_L(R)_ = 600 meV and Γ_B_ = 10 meV, which are within the acceptable range.^13,46,47^ The temperature is assumed to be 300 K. We calculate the current at the *i*th probe with
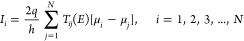
4where *T*_*ij*_ = Γ_*i*_*G*^r^Γ_*j*_*G*^a^ is the transmission probability between the *i*th and *j*th probes, *G*^a^ = (*G*^r^)^†^ is the advanced Green’s function. *N* is the total number of atoms in the system. Next, we express the chemical potential (μ) of the *i*th decoherence probe with the following equation, where *N*_b_ represents the number of Büttiker probes (atoms excluding the contact atoms)
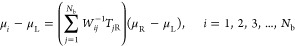
5

Here,  is the inverse of *W*_*ij*_ = [(1 – *R*_*ii*_)δ_*ij*_ – *T*_*ij*_(1 – δ_*ij*_)], where *R*_*ii*_ is the reflection probability at probe *i* and is given by . Next, we compute the current at the left contact as follows:
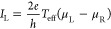
6

Here, we calculate the effective transmission with the following equations:
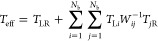
7

In eq 7, *T*_LR_ is the direct transmission between the left and right electrodes. The second term is the decoherence contribution to the transmission via decoherence probes. From eq 6, the zero bias conductance can be approximated as *G* = *G*_0_*T*_eff_, where the quantum of conductance .

We compute the density of states (DOS) values for each atom (*m*) with the following equation:
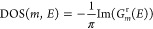
8

For the 2D DOS plots, we sum up the DOS values of each atom for the corresponding nucleobase and energy.

## Results and Discussion

**Figure 2:**
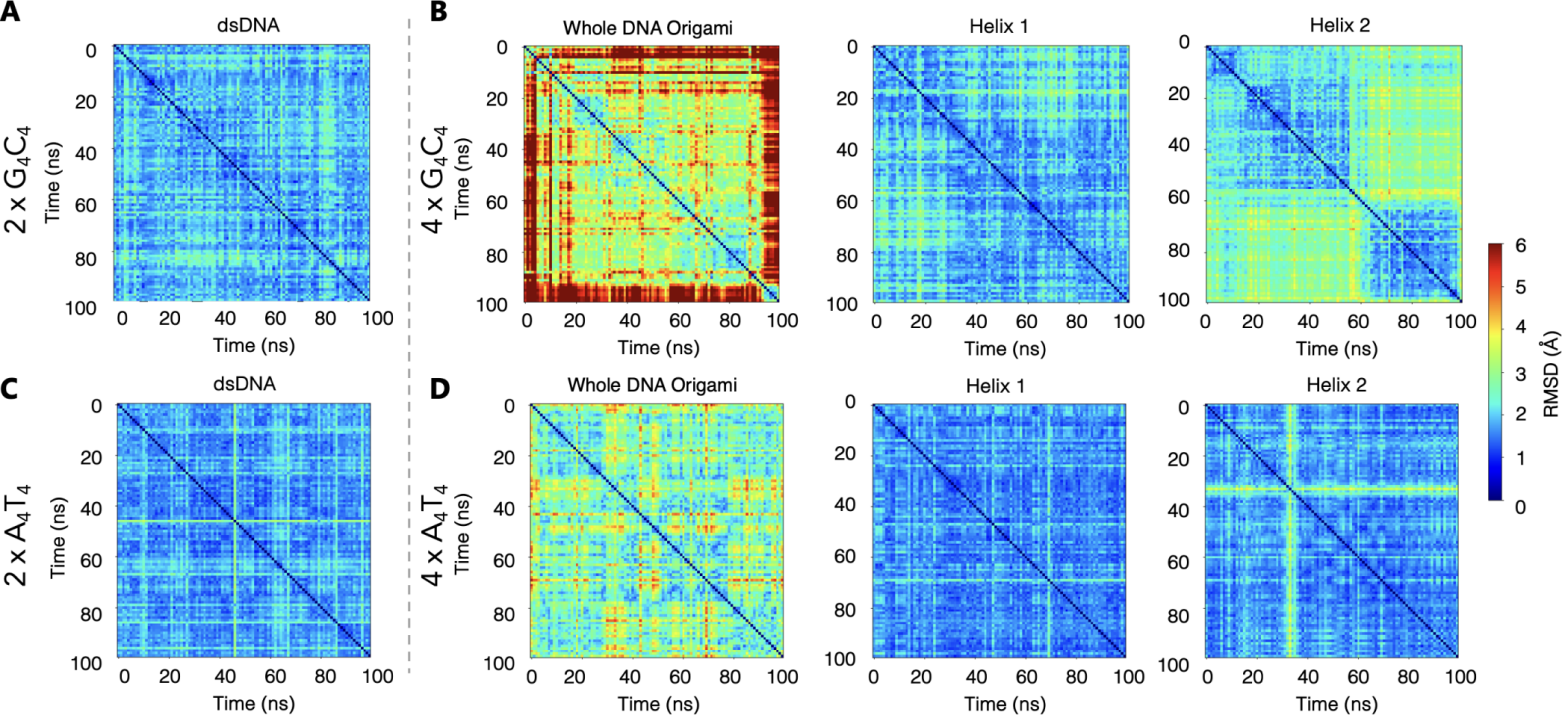
Pairwise RMSD analysis for (A) 4 × G_4_C_4_ DNA origami, (B) 4 × G_4_C_4_ dsDNA, (C) 4 × A_4_T_4_ DNA origami, and (D) 4 × A_4_T_4_ dsDNA. The graphs show the variation of RMSD between every conformation saved at 1 ns time intervals. Higher values of RMSD indicate a greater structural change between the two conformations.

To understand the electronic properties of double helix DNA origami structures and the differences/similarities with their dsDNA counterparts, we first perform MD simulations to obtain the molecular conformations and assess the structural stability. Then, we analyze the MD trajectories and calculate root-mean-square deviation (RMSD) (Figure S2) and generate pairwise RMSD plots (Figure 2), which enable us to understand the conformational changes and structural stability in MD simulations. The pairwise RMSD plots illustrate the structural changes within the dsDNA (Figure 2A,C) and DNA origami together with the individual helices forming the DNA origami structure (Figure 2B,D). Pairwise RMSD analysis (Figure 2) together with RMSD plots (Figure S2) indicate that fluctuations in DNA origami are higher than those in dsDNA for both 4 × G_4_C_4_ and 4 × A_4_T_4_ sequences: DNA origami has two double-stranded helices and thus has interhelical interactions (attractions and repulsions between the helices), which do not govern as uniquely as the intrahelical interactions (i.e., well-defined H-bonds). As seen in Figure S2, when individual helices are considered (named as helix 1 and helix 2), the amplitude of the fluctuations is around 2 Å, indicating intrahelical stability after a few nanoseconds. This fluctuation is similar to what we observe in dsDNA alone. On the other hand, when we consider the whole origami structures, the amplitudes are around 4 Å for 4 × G_4_C_4_, indicating lower stability due to the repulsions between the two helices, i.e., one helix rotates relative to the other around the crossover. This is exhibited by two distinct conformations at different time intervals: one between 0 and 60 ns and the other between 60 and 100 ns for the 4 × G_4_C_4_ sequence (see helix 2 of Figure 2B). We did not observe the same interhelical rotation for 4 × A_4_T_4_ case (amplitude of the fluctuations is around 3 Å) over the 100 ns simulation time.

We then perform the root-mean-square fluctuation (RMSF) calculation for each atom to analyze the rigidity within the molecular structures (Figure S3). This analysis demonstrates that the fluctuations observed in both dsDNA and origami mostly happen in the terminal regions rather than the central parts. Furthermore, when compared with dsDNA, DNA origamis (4 × G_4_C_4_ and 4 × A_4_T_4_) exhibit higher RMSF values through the central regions of the structures (near the crossover region). These observations are also corroborated with the temporal analysis of the number of hydrogen bonds (H-bonds) (Figure S4). We note that for 4 × G_4_C_4_ DNA origami, at around 60 ns, there is a significant drop in the number of H-bonds for helix 2, indicating the previously explained interhelical rotation.

The analysis of the electronic properties of each structure via DFT calculations is computationally too costly. Thus, we perform clustering (see the Theoretical Methods section) on all the conformations sampled during the MD simulations (Figure S5) to choose representative structures. Once we cluster the structures into groups, we then calculate the structure at the geometric center of all the structures within the most populous clusters and select it as the representative structure. To relax the selected structures, we perform energy minimization before the DFT calculations. The three-dimensional images of these representative structures are given in Figures S6 and 1. As we discussed above, 4 × G_4_C_4_ DNA origami exhibits another very distinct conformation after the aforementioned interhelical rotation, captured as cluster 4. Thus, this representative structure of the distinct cluster has also been chosen for further analysis (4 × G_4_C_4_ C2 in Figure S6). As indicated by Figure S7, individual helices of both DNA origami differ from the dsDNA around 1.5 Å thus we state that the individual helices closely resemble the representative dsDNA conformation.

**Figure 3:**
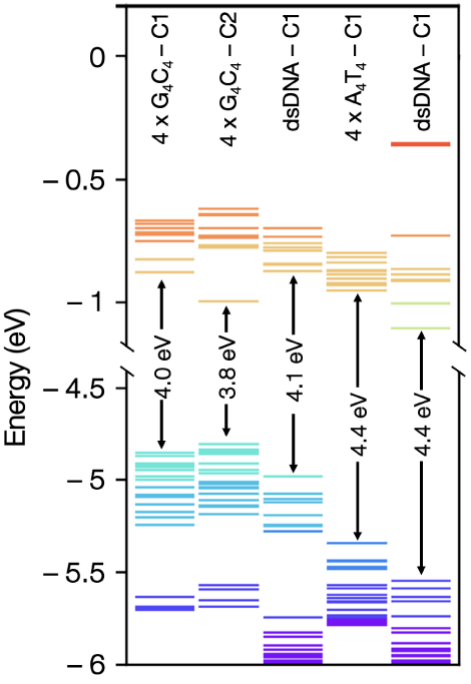
Energy band diagram for 20 occupied and 10 unoccupied molecular orbitals in the vicinity of the HOMO–LUMO gap for each structure.

Next, we perform DFT calculations on all the structures chosen, to obtain the density of states (DOS) along the molecules and the energy levels. Figure 3 shows the first 20 occupied (HOMO levels) and 10 unoccupied (LUMO levels) energy levels of each structure. We notice that in 4 × G_4_C_4_ the energies of occupied orbitals shifted toward higher energies, while the energies of unoccupied orbitals remain mostly unchanged (C1) or shifted toward lower energies (C2) compared to dsDNA (Figure 3). However, in 4 × A_4_T_4_ case, the energies of both the occupied and unoccupied orbitals shifted toward higher energies compared to its dsDNA equivalent (Figure 3). As a result, the band gap is reduced in the 4 × G_4_C_4_ DNA origami case, while remained same in the 4 × A_4_T_4_ DNA origami case compared to their ds-DNA counterparts.

**Figure 4:**
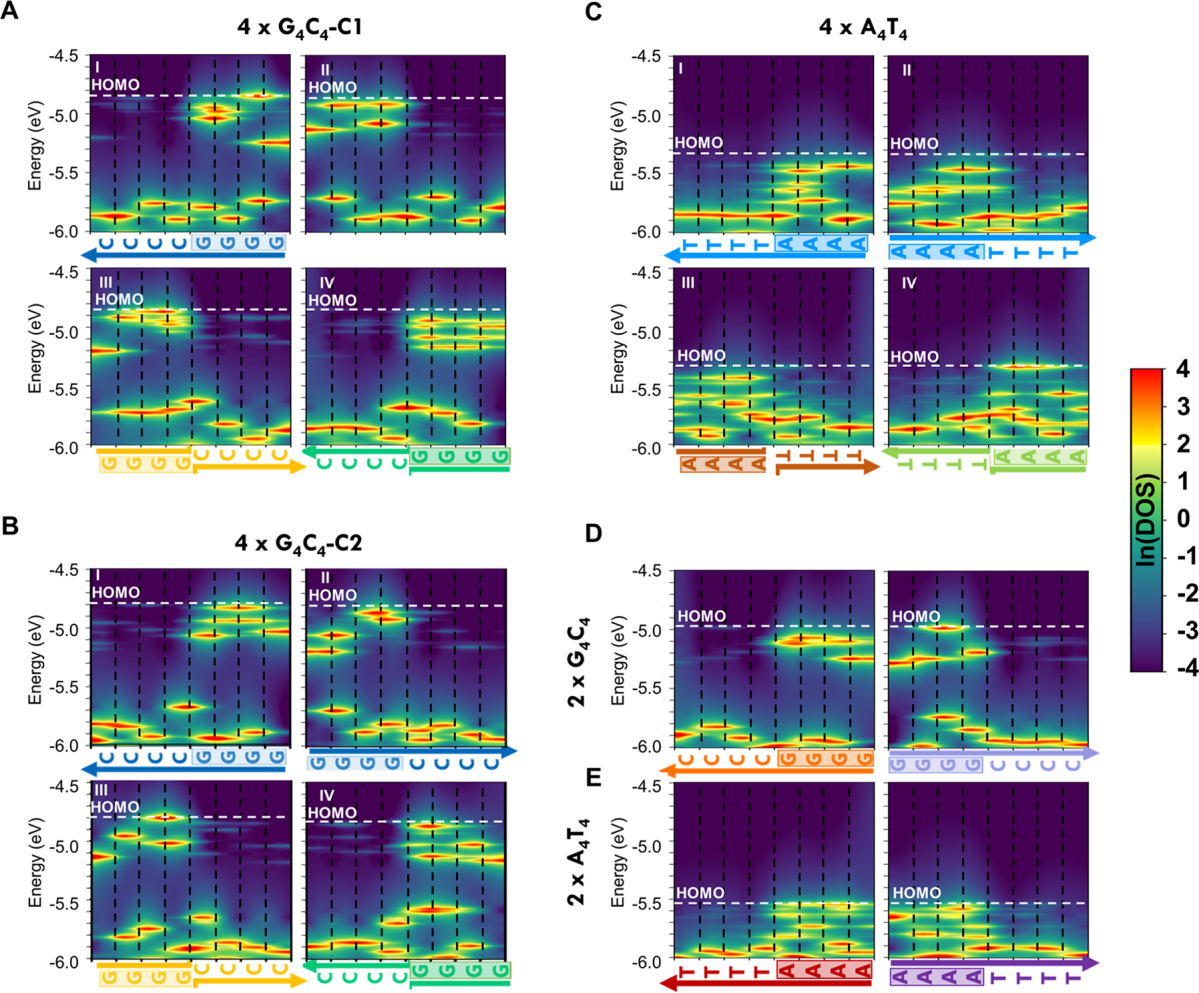
Density of states (DOS) on each strand of DNA origami and dsDNA structure of 4 × G_4_C_4_ and 4 × A_4_T_4_ sequence cases. DOS for (A) DNA origami 4 × G_4_C_4_–C1, (B) DNA origami 4 × G_4_C_4_–C2, (C) dsDNA 4 × A_4_T_4_, (D) dsDNA 2 × G_4_C_4_, and (E) dsDNA 2 × A_4_T_4_ case.

In Figure 4A–E, we plot the DOS as a function of energy along individual strands of the molecules to analyze the charge transmission contributions of each occupied molecular orbital. In these plots, the DOS resulting from the occupied molecular orbitals appears not localized on one single base but broadened into neighboring bases. We observe that the DOS values residing on guanines have higher energy levels compared to cytosines in both 4 × G_4_C_4_ DNA origami and ds-DNA case. Besides, the DOS values residing on adenines have higher energy levels compared to thymines in both 4 × A_4_T_4_ DNA origami and ds-DNA case. Another interesting observation is that the energy levels residing on guanines are almost 0.5 eV above the cytosines, forming an extra intra band gap within the HOMO regions. On the contrary, the energy levels from thymines and adenines show rather a continuum in HOMO regions. Next, we observe that both in dsDNA and DNA origami structures, the HOMO and HOMO-1, which are the primary orbitals contributing to the charge transmission, are localized on different strands in GC sequence and are spread between strands in AT sequence (Figure S8 orbitals). The energy separation between these two orbitals varies between DNA origami and dsDNA. For example, the 4 × G_4_C_4_ DNA origami’s (C1) HOMO is on the 5′-GG of the strand I, and HOMO-1 is on strand III; the energy separation between them is 19 meV (Figure 4A). Similarly, DNA origami structure of the 4 × A_4_T_4_ sequence has the HOMO on strand IV, while HOMO-1 is on strand III (Figure 4C). However, 4 × A_4_T_4_ sequence’s HOMO and HOMO-1 energy levels are 92 meV apart, which is significantly more than that of 4 × G_4_C_4_ sequences. In 4 × G_4_C_4_ ds-DNA, the HOMO and HOMO-1 are localized on different strands, and the energy separation between the two molecular orbitals is 100 meV. In contrast, in dsDNA AT case, the energy separation between the two orbitals is 38 meV, and HOMO-1 orbitals are localized on both strands. While in 4 × G_4_C_4_ sequence, as the structure goes from dsDNA to DNA origami, the energy level separation between the first two HOMO orbitals decreases by ∼80% (for both C1 and C2), it increases in the 4 × A_4_T_4_ case by 58%. These results suggest that in 4 × G_4_C_4_ sequences, while the DNA origami could transfer charge more efficiently than dsDNA, in 4 × A_4_T_4_ sequence, it is the other way around. Furthermore, we notice that crossover regions do not have significant DOS in the vicinity of the HOMO in either GC or AT sequences. As previous studies^13^ reported, in DNA, the energy levels near the Fermi level of gold electrodes are located on the nucleobases rather than backbone atoms. On the other hand, the backbone type (i.e., peptide nucleic acids) is found to influence the overall charge transmission in DNA molecules.^48^ Therefore, we suggest that functionalization of the DNA backbone in origami structures may alter the availability of states in the crossover regions near the HOMO and thus change the conductance. However, this falls beyond the scope of this study and should be looked at in the future studies.

**Figure 5:**
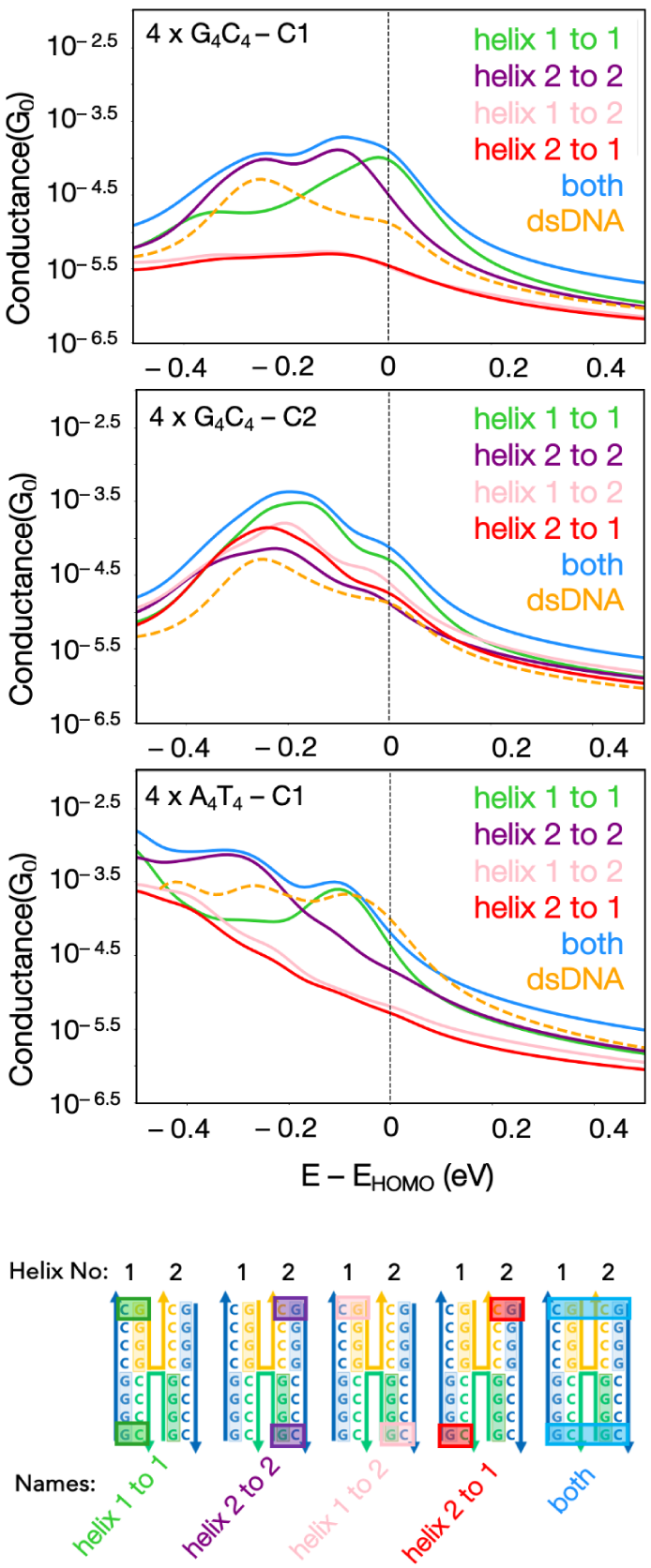
Conductance plots for 4 × G_4_C_4_ DNA origami C1 and C2, 4 × A_4_T_4_ DNA origami, and their counterpart dsDNAs. DNA origami electrode contacts are made from helix 1 to 1, 2 to 2, 1 to 2, 2 to 1, and from both helices.

Next, we obtain the Hamiltonian matrices for the structures and explore the electronic properties using the Green’s function method. The Hamiltonian matrices are obtained with B3LYP/6-31g(d,p) level calculation using GAUSSIAN 16 software package (see the Theoretical Methods section for details). In this approach, we use broadening matrices instead of modeling the gold electrodes and the linker molecules explicitly, and it has been shown to resemble experimental results effectively in different studies.^12,20,22,49,50^ Here, the electrodes are assumed to make contact only with the terminal base pair atoms. Thus, we select contact atoms from terminal regions as illustrated in Figure 5. The atomic representations of the contacts and the linkers are also given in Figures S9 and S10, respectively. These different contact positions are named as follows for clarity: contacts at both terminal base pairs of the individual helices of DNA origami structure as *helix* 1 *to* 1 and *helix* 2 *to* 2; contacts at both terminal base pairs of DNA origami helices *both*; contacts at the ends of the different terminal base pairs of the individual DNA origami helices as *helix* 1 *to* 2 and *helix* 2 *to* 1. To compare DNA origami with dsDNA counterparts, we align all the HOMO values of the structures as shown in Figure 5. We observe that *helix* 1 *to* 1, *helix* 2 *to* 2, and *both* cases clearly have higher conductance relative to *helix* 1 *to* 2 and *helix* 2 *to* 1 in 4 × G_4_C_4_–C1 and 4 × A_4_T_4_ cases. On the other hand, both *helix* 1 *to* 2 and *helix* 2 *to* 1 have significantly lower conductance values in 4 × G_4_C_4_–C1 and 4 × A_4_T_4_ cases. The underlying physical reason can be explained with the molecular orbital locations (Figure S8).

As previously mentioned, the crossover regions do not have available states close to the HOMO of the system for 4 × G_4_C_4_ C1, C2 and 4 × A_4_T_4_ conformations. Since, in both *helix* 1 *to* 2 and *helix* 2 *to* 1 cases, the charge carriers need to travel along the crossover region from one contact to another, backbone atoms in the crossover region act as an energy barrier for the system, therefore resulting in poorer conductance. Next, we compare DNA origami structures with their dsDNA counterparts. If the Fermi energy is at the HOMO for each case (or the HOMO plus a few hundred meV), the conductance of dsDNA is 3 and 10 times higher than *helix* 1 *to* 2 and *helix* 2 *to* 1 cases in 4 × G_4_C_4_ C1 and 4 × A_4_T_4_ cases, respectively. Furthermore, if the contacts are made on a single helix of DNA origami (helix 1 to 1 and helix 2 to 2), we observe that while 4 × G_4_C_4_ C1 has higher conductance, 4 × A_4_T_4_ has slightly lower conductance than their dsDNA counterpart.

**Figure 6:**
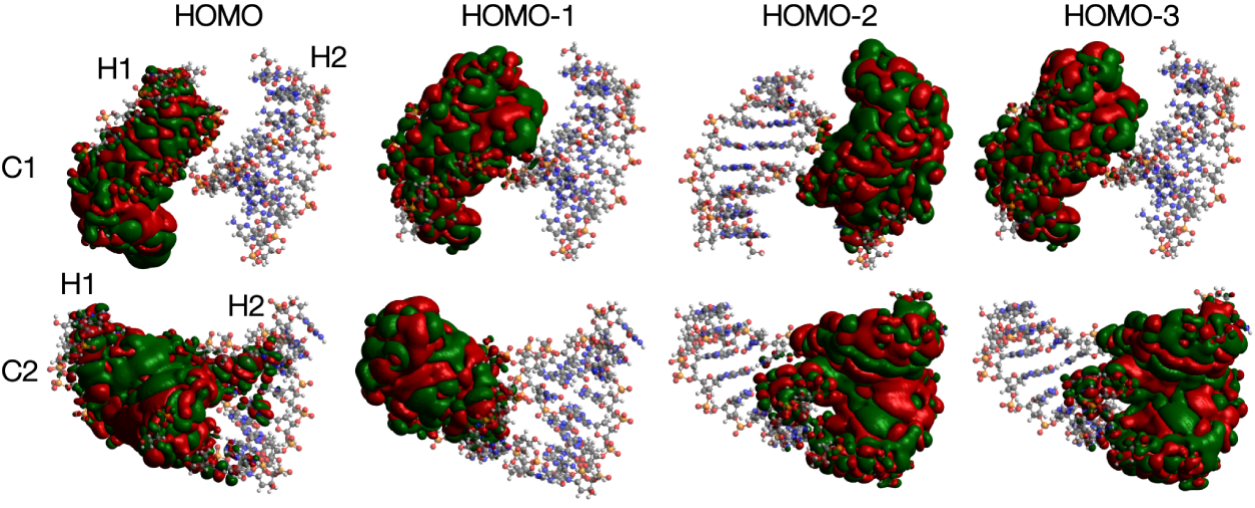
HOMO to HOMO-3 molecular orbitals projected on the different representative conformations of 4 × G_4_C_4_ sequence with iso value = 0.0002.

On the other hand, 4 × G_4_C_4_–C2 conformation shows a different trend than C1. In this case, helix 1 to 2 and helix 2 to 1 both have comparable conductance values with helix 1 to 1 and its dsDNA counterpart. When compared with C1, C2 shows higher conductance values for helix 1 to 2 and helix 2 to 1 cases. We investigate the reason behind this observation via spatial locations of the molecular orbitals. As shown in Figure S6, the terminal bases of helix 2 are closer to helix 1 due to the relative rotations of the two helices. This results in spreading molecular orbitals between the two helices as demonstrated in Figure 6. While in the C1 conformation, molecular orbitals are distinctly located on either helix 1 or 2, in C2 conformation, the orbitals in helix 1 spread toward helix 2.

## Conclusions

We investigate the electronic properties of DNA origami structures with 4 × G_4_C_4_ and 4 × A_4_T_4_ sequences. We use molecular dynamics simulations, quantum mechanical calculations, and Green’s function-based charge transport calculations to understand the electronic band structures, density of states, and molecular orbital distributions along the molecules. Our results demonstrated that DNA origami structures can have a higher (4 × G_4_C_4_ sequence) or similar (4 × A_4_T_4_ sequence) density of states compared to dsDNA analogues. While GC sequence (DNA origami) can have a lower energy band gap compared to its dsDNA counterpart, AT sequence can have the same bandgap value as the dsDNA. We reported that the crossover regions may have a low density of states in the vicinity of the HOMO because of the presence of backbone atom. We think that these regions act as an energy barrier and unless there’s another interaction (such as nucleobases come proximity to each other) between the two helices, molecular orbitals reside either on helix 1 or helix 2 and spatially separated. The position of the electrode contacts on the separate helices revealed that, depending on the molecular orbital distribution along the structure, helices can have different conductance values even if they consist of the same sequence. These results show that DNA origami’s electronic properties can be tuned with the sequence and the selection of electrode position. These parameters can be used to broaden DNA origami applications in molecular electronics and pave the way for DNA origami-based electronics.

## Supporting Information Available

Molecular dynamics trajectory and structural analysis; example DNA origami structure and a crossover region (Figure S1); RMSD vs time plots for both DNA origami and ds-DNA structure calculated from 100 ns MD trajectories (Figure S2); RMSF values for each atom calculated and averaged using every conformation in 100 ns MD simulation (Figure S3); temporal change in hydrogen bond during the simulation (Figure S4); different clusters encountered in the MD simulations for each sequence (Figure S5); representative conformations chosen for DNA origami structures (Figure S6); structural comparison of the individual helices of the representative DNA origami conformations and dsDNA used for DFT calculations (Figure S7); molecular orbitals for all structures (Figure S8); atomic representation of the contact atoms used in the conductance calculations (Figure S9); illustrative representation of the DNA molecules, gold electrodes, and thiol linkers as the terminal regions used in the conductance calculations (Figure S10) (PDF)

## Abbreviations

DNA: deoxyribonucleic Acid
MD: molecular dynamics
DFT: density functional theory
DOS: density of states
HOMO: highest occupied molecular orbital
LUMO: lowest unoccupied molecular orbitals
