# Supplementary material for: Electronic Properties of DNA Origami Nanostructures Revealed by In Silico Calculations
Source: J Phys Chem B. 2024 May 7;128(19):4646–54. doi: 10.1021/acs.jpcb.4c00445 (PMC11103695; doi:10.1021/acs.jpcb.4c00445)
Supplement: Supplementary file 1 — jp4c00445_si_001.pdf [file jp4c00445_si_001.pdf]

Supplementary Information

Electronic Properties of DNA Origami Nanostructure Revealed by in Silico Calculations

Busra Demir[1,2,3], *Caglanaz Akin Gultakti*[1,2], *Zeynep Koker*[2], *M.P. Anantram*[3,*], *Ersin Emre Oren*[1,2,*]

[1] Department of Materials Science & Nanotechnology Engineering, TOBB University of Economics and Technology, Ankara 06560, Türkiye.

[2] Bionanodesign Laboratory, Department of Biomedical Engineering, TOBB University of Economics and Technology, Ankara 06560, Türkiye.

[3] Department of Electrical and Computer Engineering, University of Washington, Seattle, WA 98115, USA

* *corresponding authors*

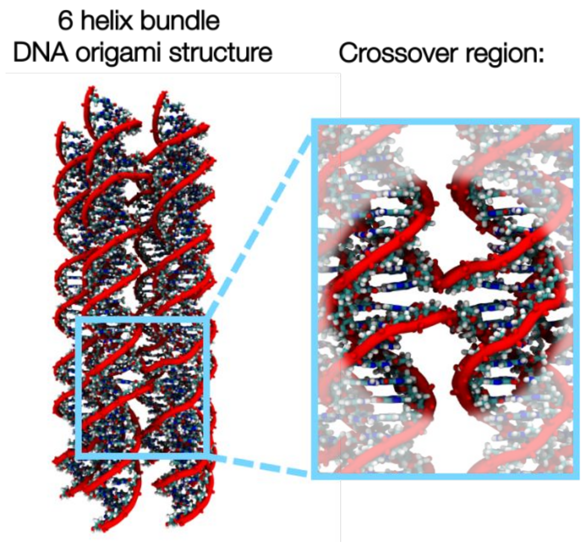

Figure S1: Example DNA origami structure and a crossover region.

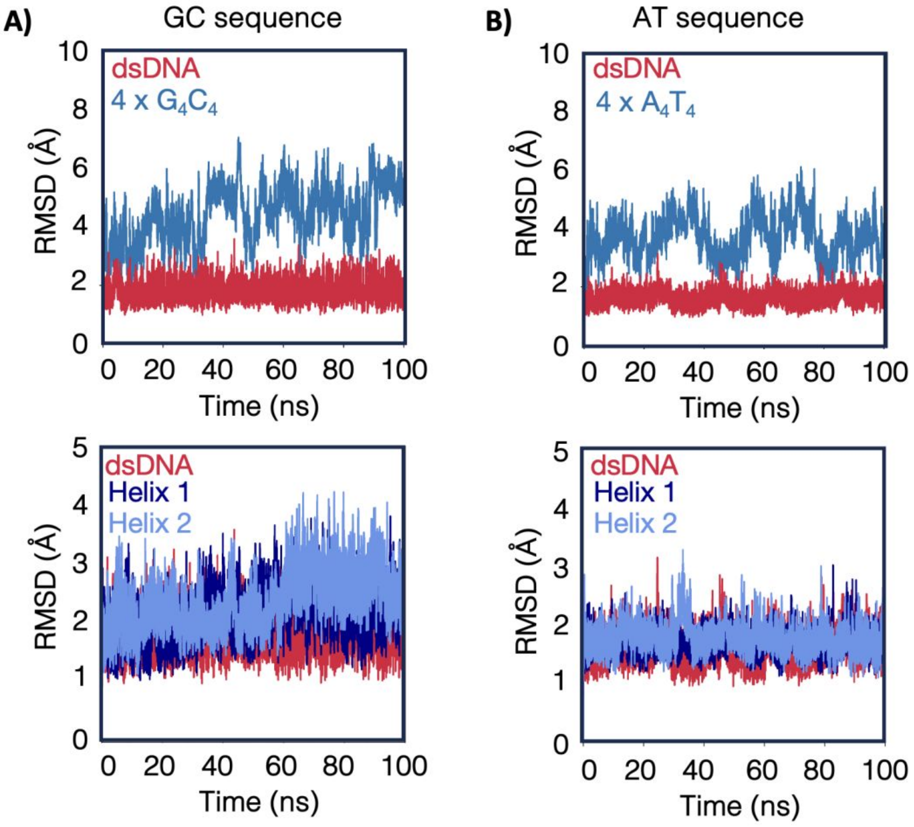

Figure S2: RMSD vs time plots for both DNA origami and ds-DNA structure calculated from 100 ns MD trajectories A) GC sequence, B) AT sequence. The RMSD is calculated with respect to the initial conformation of corresponding structures after energy minimization.

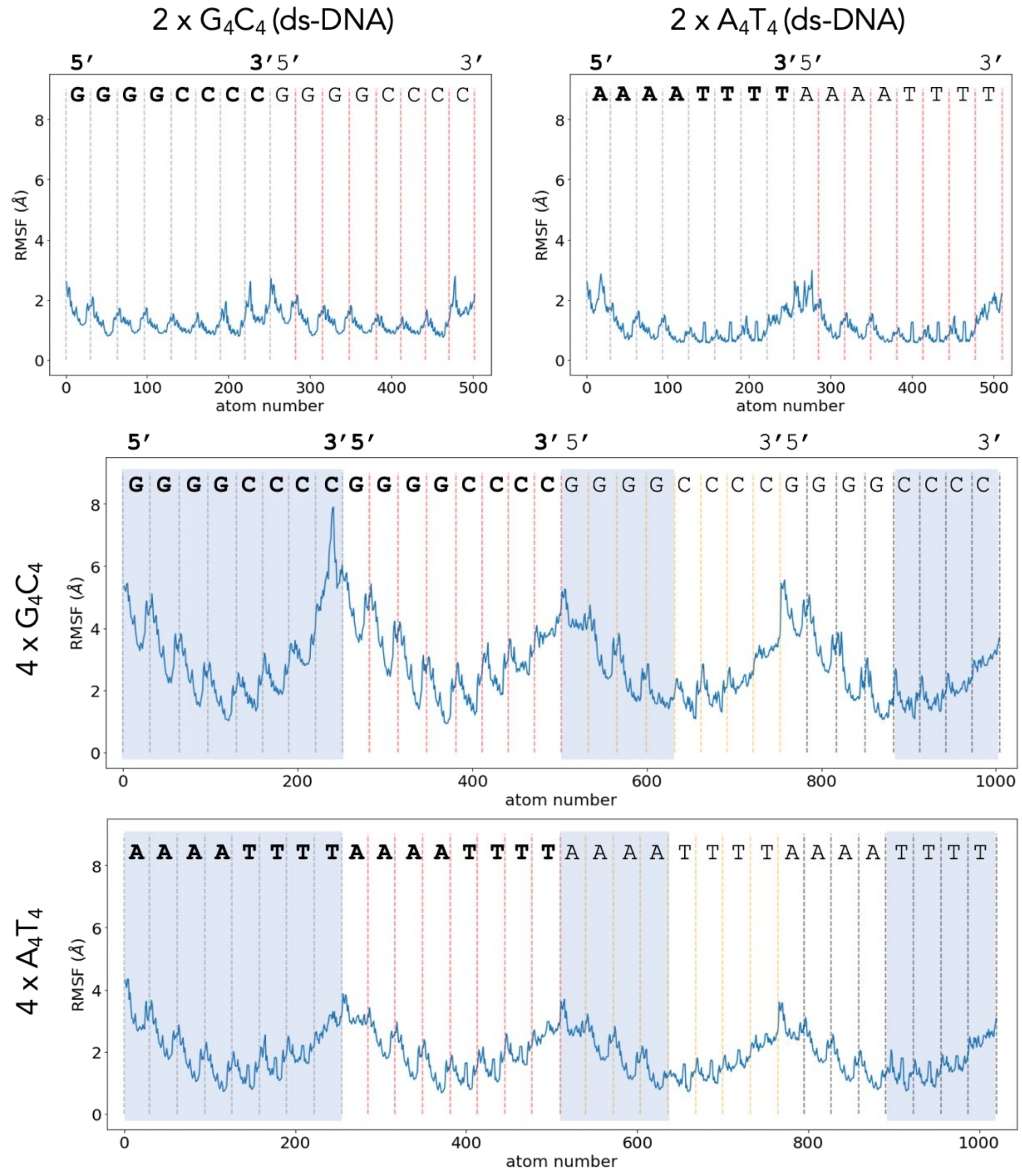

Figure S3: RMSF values for each atom calculated and averaged using every conformation in 100 ns MD simulation. The top two plots correspond to the results for DNA origami structures and the last two plots display results for ds-DNA structures both with GC and AT rich sequence. The 5'

and 3' notations above each plot represent the terminal bases of each structure and the bold and normal font types used to distinguish between different strands. The highlighted regions in the above plots correspond to the residues associated with helix 2, as defined in the main manuscript.

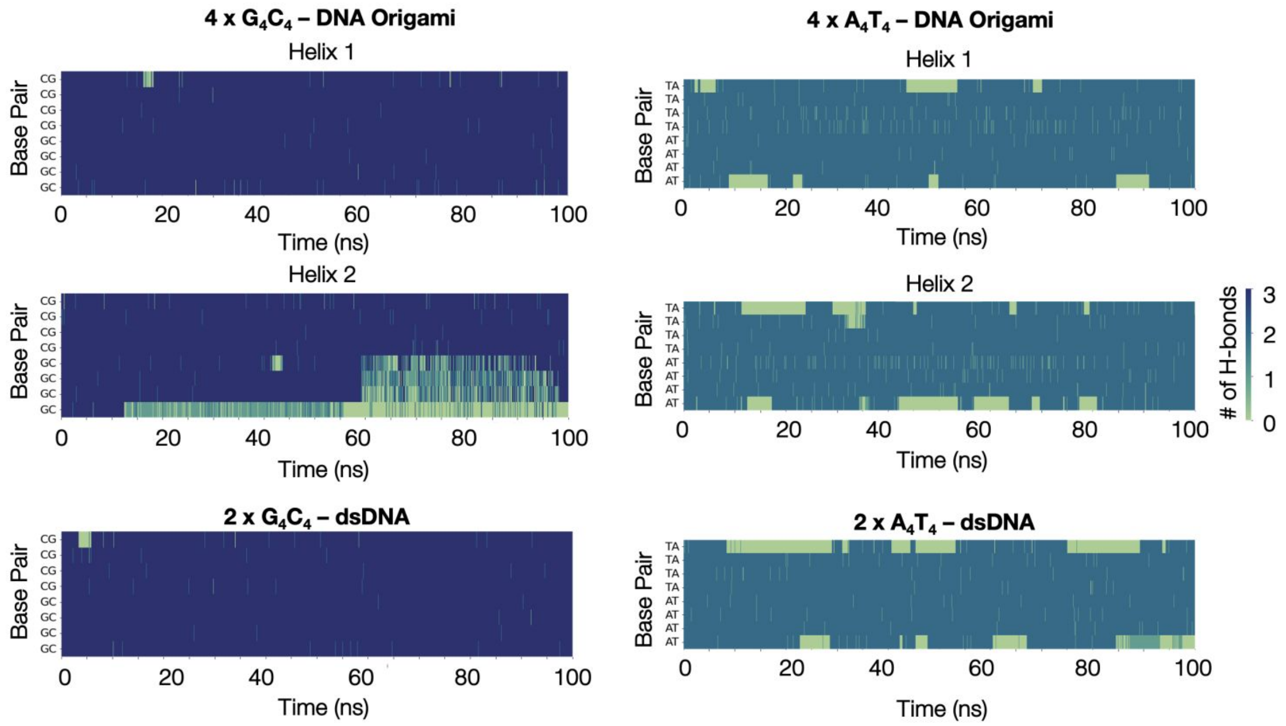

Figure S4: Temporal change in hydrogen bond during the simulation.

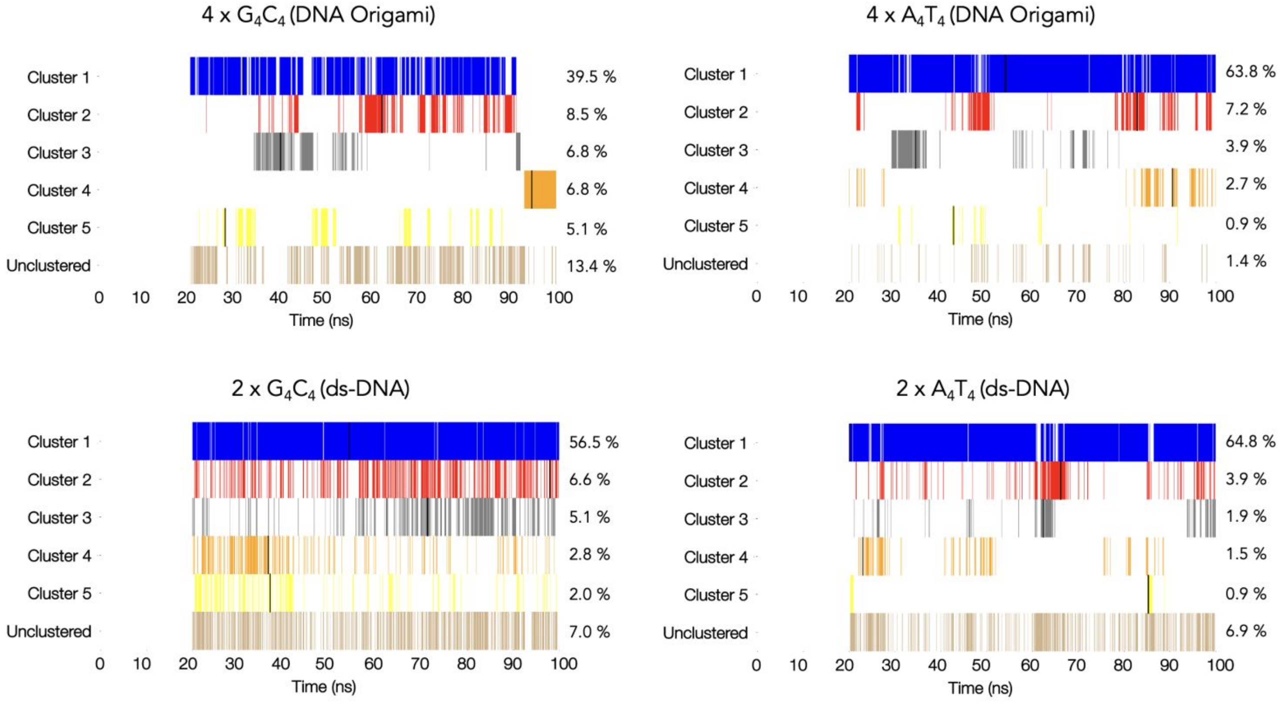

Figure S5: Different clusters encountered in the MD simulations for each sequence. Percentages represent the cluster density calculated by (number of conformations in the cluster / total number of conformations).

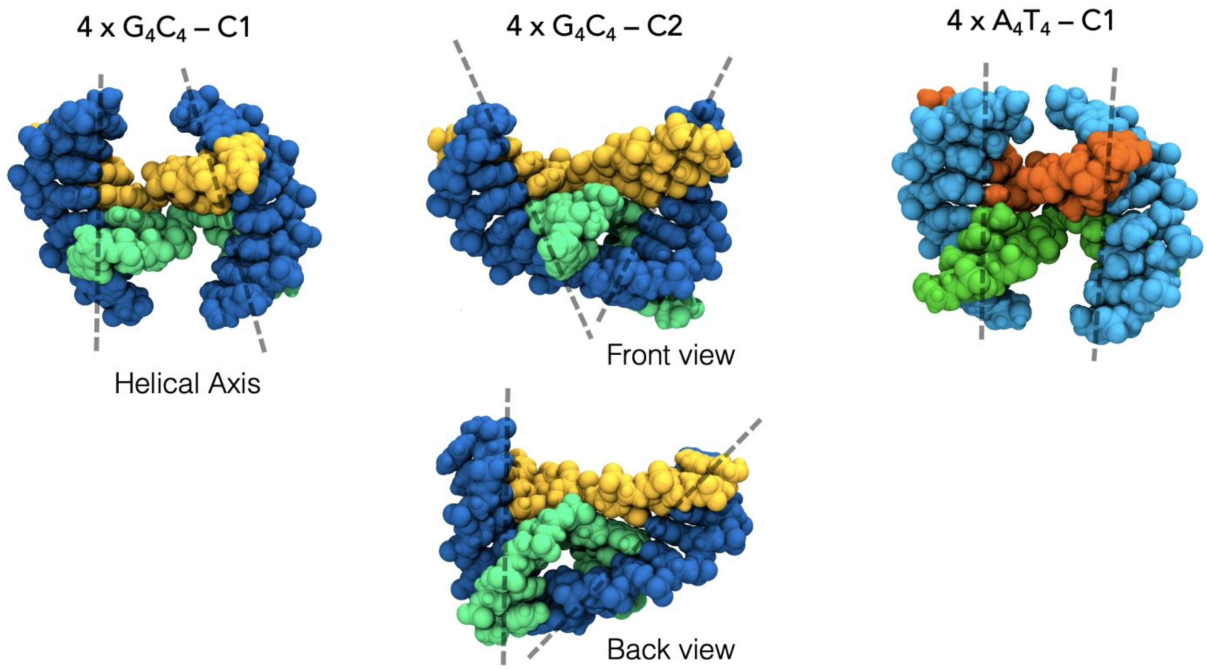

Figure S6: Representative conformations chosen for DNA origami structures both for $4xG_4C_4$ and $4xA_4T_4$ cases.

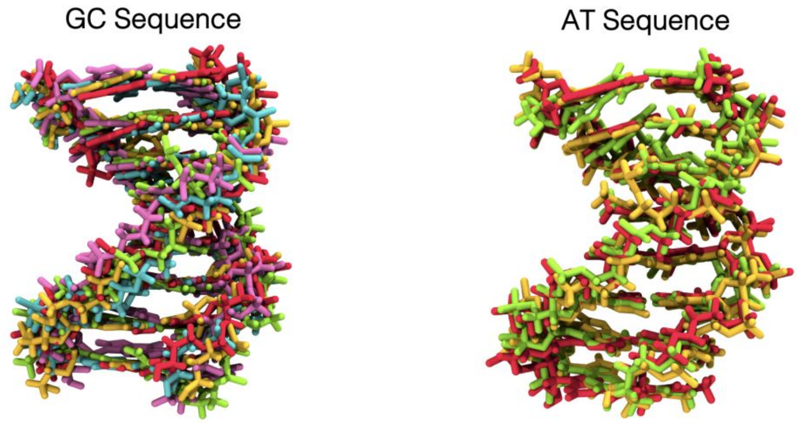

		RMSD to dsDNA (Å)
C1	Helix 1	1.614
	Helix 2	2.096
C2	Helix 1	1.902
	Helix 2	2.178

	RMSD to dsDNA (Å)
Helix 1	1.272
Helix 2	1.294

Figure S7: Structural comparison of the individual helices of the representative DNA origami conformations and dsDNA used for DFT calculations.

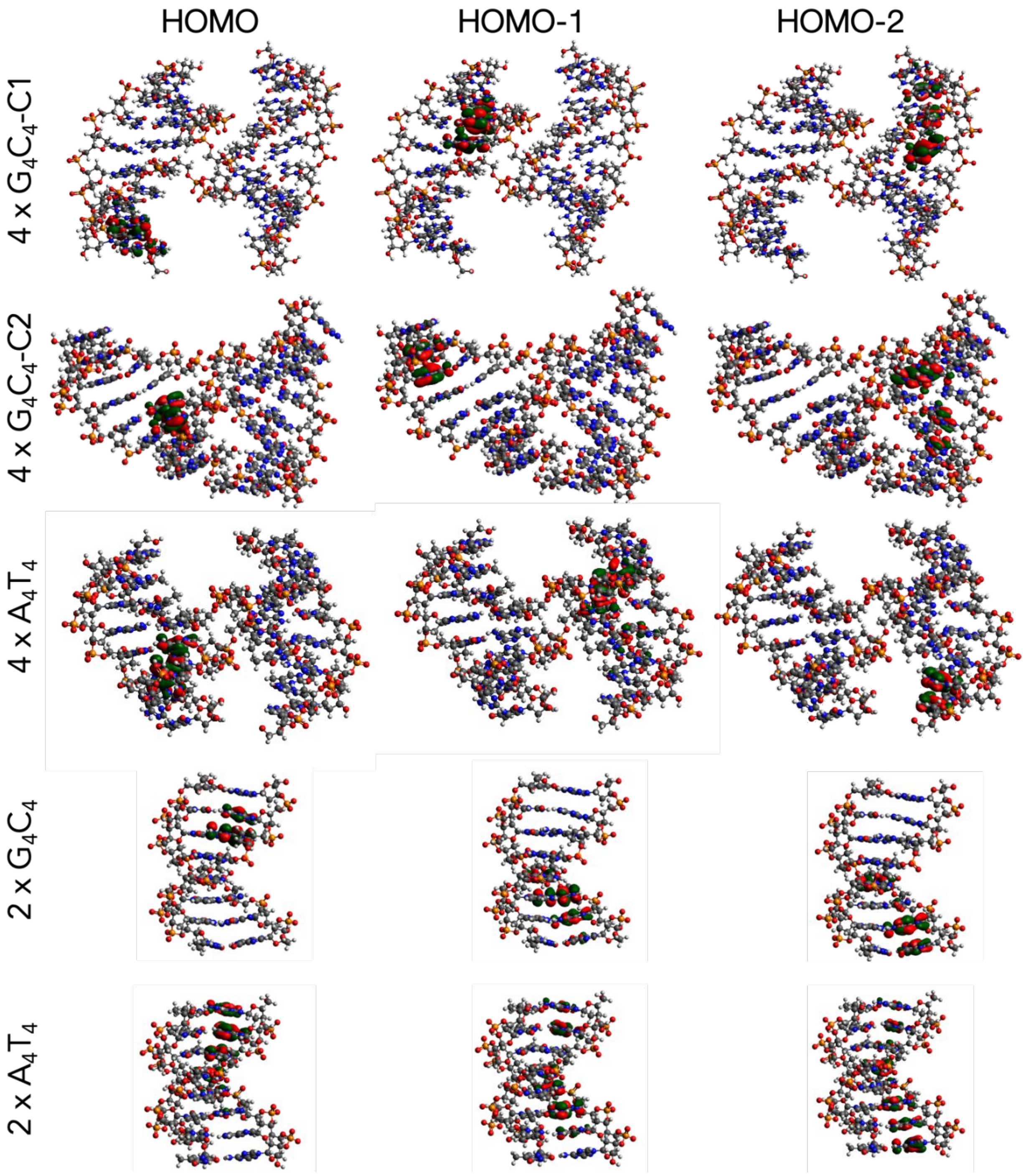

Figure S8: Molecular orbitals for all structures with iso value= 0.02.

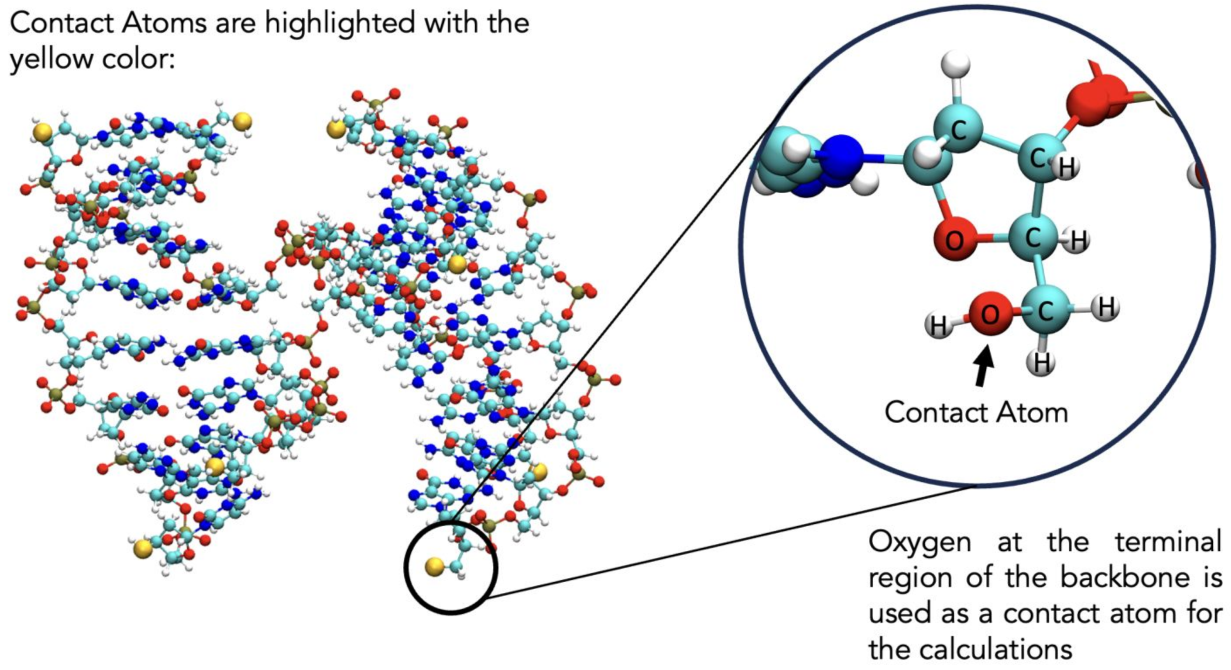

Figure S9: Atomic representation of the contact atoms used in the conductance calculations.

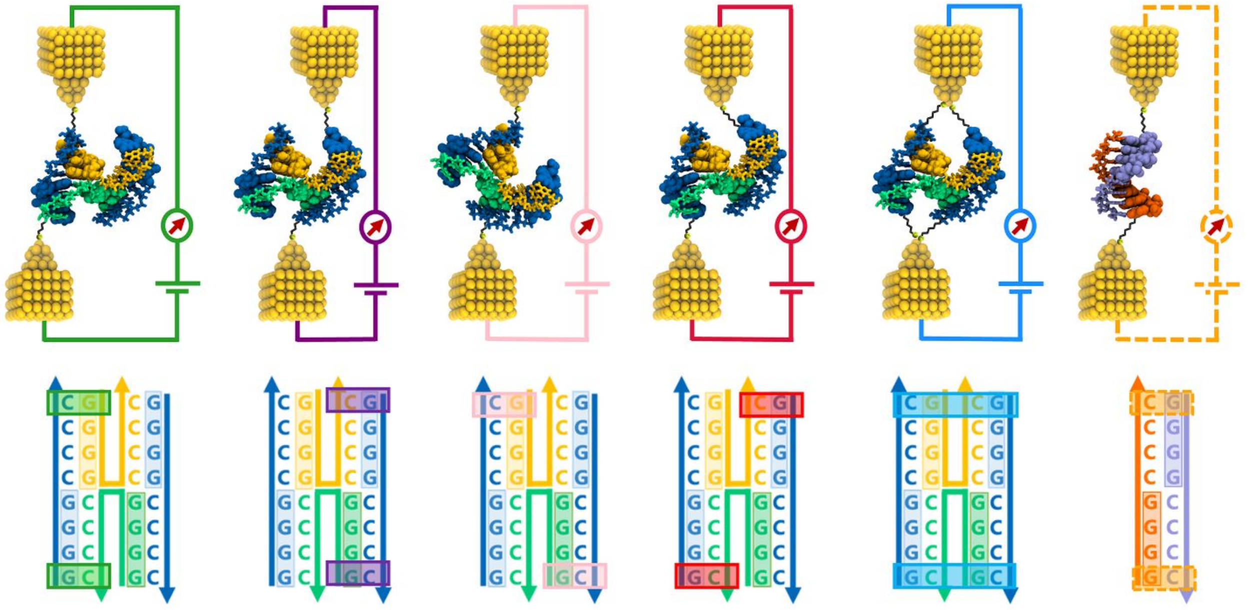

Figure S10: Illustrative representation of the DNA molecules, gold electrodes and thiol linkers as the terminal regions used in the conductance calculations.
